# p53 Gene (NY-CO-13) Levels in Patients with Chronic Myeloid Leukemia: The Role of Imatinib and Nilotinib

**DOI:** 10.3390/diseases6010013

**Published:** 2018-01-25

**Authors:** Hayder M. Al-kuraishy, Ali I. Al-Gareeb, Ali K. Al-Buhadilly

**Affiliations:** Department Of Pharmacology, Toxicology, and Medicine, College of Medicine Al-mustansiriyah University, P.O. Box 14132 Baghdad, Iraq; dr.alialgareeb78@yahoo.com (A.I.A.-G.); alikadm1977@yahoo.com (A.K.A.-B.)

**Keywords:** nilotinib, imatinib, p53, CML

## Abstract

The p53 gene is also known as tumor suppressor p53. The main functions of the p53 gene are an anticancer effect and cellular genomic stability via various pathways including activation of DNA repair, induction of apoptosis, and arresting of cell growth at the G1/S phase. Normally, the p53 gene is inactivated by mouse double minute 2 proteins (mdm2), but it is activated in chronic myeloid leukemia (CML). Tyrosine kinase inhibitors are effective chemotherapeutic agents in the management of CML. The purpose of the present study was to evaluate the differential effect of imatinib and nilotinib on p53 gene serum levels in patients with CML. A total number of 60 patients with chronic myeloid leukemia with ages ranging from 47 to 59 years were recruited from the Iraqi Hematology Center. They started with tyrosine kinase inhibitors as first-line chemotherapy. They were divided into two groups—Group A, 29 patients treated with imatinib and Group B, 31 patients treated with nilotinib—and compared with 28 healthy subjects for evaluation p53 serum levels regarding the selective effect of either imatinib or nilotinib. There were significantly (*p* < 0.01) high p53 gene serum levels in patients with CML (2.135 ± 1.44 ng/mL) compared to the control (0.142 ± 0.11 ng/mL). Patients with CML that were treated with either imatinib or nilotinib showed insignificant differences in most of the hematological profile (*p* > 0.05) whereas, p53 serum levels were high (3.22 ± 1.99 ng/mL) in nilotinib-treated patients and relatively low (1.18 ± 0.19 ng/mL) in imatinib-treated patients (*p* = 0.0001). Conclusions: Nilotinib is more effective than imatinib in raising p53 serum levels in patients with chronic myeloid leukemia.

## 1. Introduction

The p53 gene is known also as tumor suppressor p53, cellular tumor antigen p53 or NY-CO-13 transforming-related protein 53 and it acts as a tumour suppressor and prevents mutation, so it is called a tumour suppressor gene and found on chromosome 17 [1]. The p53 gene plays an important role in the prevention of cancer initiation and formation, so most of the p53 gene is a mutated gene (>50%) [2]. The main functions of the p53 gene are an anticancer effect and cellular genomic stability via various pathways including activation of DNA repair, induction of apoptosis and arresting of cell growth at the G1/S phase [3]. 

Normally, the p53 gene is inactivated by mouse double minute 2 proteins (mdm2),so the activated form of this antigen is low; however, when there are activators like cell-cycle abnormality, hypoxia, and DNA damage, this leads to the dissociation of mdm2 and then the p53 gene will be activated causing DNA repair and induction of apoptosis [4]. 

Furthermore, when p53 gene is stimulated it binds to DNA and stimulates several genes like p21, micro RNA, and CDK2 that are involved in impeding cell division through inhibition of kinase activity [5]. 

Moreover, the p53 gene is protected from oncogenic insults by a specific protein called ubiquitin protein, which is also required for activation and stabilization of p53 gene [6]. Consequently, activation of this gene may be an optional way for management of different types of malignancy, but, this is not recommended due to the acceleration of premature aging. The pharmacological reactivation of endogenous p53 gene was tried when gendicine was used for squamous cell carcinoma [7].

Chronic myeloid leukemia (CML) is a hematological disease caused by bone marrow proliferative disorders. In CML, there is 5–15% association with Philadelphia chromosome, which results from a reciprocal translocation between chromosome 9 and 22 resulting in the formation of a BCR-ABL fusion protein thay encodes for the tyrosine kinase pathway, and this causes uncontrolled cell division, inhibition of DNA repairs, genomic instability, and the commencement of CML with induction of blast crisis. These changes might be due to an inhibition of p53 activity [8]. Furthermore, BCR-ABL fusion protein 210 kDa is illustrated with 90% of CML, which induces malignant transformation through inhibition of cell adhesion, apoptosis, and differentiation [9].

Tyrosine kinase inhibitors are effective chemotherapeutic agents in the management of CML. They are also called as tyrphostins. They include imatinib, which is effective against different malignancies includes CML, mastocytosis and myelodysplastic syndrome. Imatinib acts through the inhibition of BCR-ABL tyrosine kinase, which induces apoptosis in cancer cell lines; it may cause different side effects including pancytopenia, heart failure, and edema [10]. On the other hand, nilotinib is a selective tyrosine kinase inhibitor that is 10–30 folds more potent than imatinib in the inhibition of BCR-ABL tyrosine kinase and mainly used for imatinib-resistant CML [11].

Therefore, the purpose of the present study was to evaluate the differential effect of imatinib and nilotinib on p53 gene serum levels in patients with chronic myeloid leukemia.

## 2. Materials and Methods

A total number of 60 patients with chronic myeloid leukemia (33 males and 27 females) with ages ranging from 47 to 59 years were recruited from the Iraqi Hematology Center. They started with tyrosine kinase inhibitors as first-line chemotherapy in addition to conservative treatments. They were divided into two groups: Group A, 29 patients (19 males + 10 females) treated with imatinib and Group B: 31 patients (16 males + 15 females) treated with nilotinib—and compared with 28 healthy subjects (18 males + 10 females) for evaluation of p53 serum levels regarding the selective effect of either imatinib tablets 200 mg/day (Imatinib mesylate tablet, Taji pharmaceutical Ltd., Mumbai, India) or nilotinib capsule 200 mg/twice daily (Tasigna capsule 200 mg, Novartis, Mumbai, India). The study was done according to the principle of the Helsinki Declaration [12].Patients gave informed written consent to their enrollment. This study was done at the Department of Clinical Pharmacology and Therapeutics in cooperation with the Iraqi Hematology Center, and was approved by the ethical committee in the College of Medicine, Al-Mustansiriyiah University, Iraq, Baghdad, 2016.

The inclusion criterion was patients with CML at the chronic phase received only tyrosine kinase inhibitors.

Exclusion criteria include patients with complications, in the blast crisis of CML, with heart failure, with chronic kidney disease, with end-stage liver disease, and in the process of combined chemotherapy (tyrosine kinase inhibitors and other chemotherapeutic agents).

Ten milliliters of venous blood were collected from each patient—8 mL for a complete blood picture and routine investigations and 2 mL for the determination of p53 serum levels in ng/mL by an ELISA kit method (Abcam, biochemicals, Cambridge, MA, USA). The principle of measurement of p53 concentrations was doneaccording to kit instructions.

### 2.1. Principle of Assessment of Serum p53 Concentration

This was done by the ELISA method, after optimization of the reaction. Microtiter plates were coated with 50 μL/well of each sample. When diluted 1:1000 at a pH of 9.6, the plates were incubated at room temperature for 2 h and washed using 0.05% PBS-T20 and then incubated at 37 °C for 1 h with 200 μL/well of the buffer solution at pH of 9.6. After washing 50 μL/well of monoclonal antibody Bp53-12, it was diluted and PBS-T20 (1:100) were added and incubated for 2 h at 37 °C. After a subsequent reaction that stopped using a stop solution of 25 μL/well (3 M NaOH), the absorbance was read at 405 nm by microplate auto reader. The cutoff value was equivalent to 0.212 ng/mL.

### 2.2. Statistical Analysis

Data analysis was done using statistical package for the social sciences SPSS (IBM, version 20.0, IBM Corp, 2012 Armonk, NY, USA). Data are presented as mean ± SD, number and percentage. Unpaired t-test was used to estimate the significance of the difference between the patients and healthy controls, regarding a *p*-value less than 0.05 as significant.

## 3. Results

Sixty (68.18%) patients and 28 (31.18%) of healthy control with a mean age 52.67 ± 5.12 years were enrolled. The duration of CML was 2.1 ± 1.33 years. A percent of 48.33% of patients were treated with imatinib, and 51.67% were treated with nilotinib. Moreover, patients with CML presented with hepatosplenomegaly as mild (51.67%), moderate (36.67%) and severe (15.0%). Philadelphia chromosome was positive in about 18.33% and negative in 81.67% of patients with CM, (Table 1).

The differences in the hematological profile revealed highly significant differences in white blood cells, platelet count, plateletcrit percentage and mean corpuscular hemoglobin concentration in patients with CML compared with control (*p* < 0.01). Additionally, RDWCV (%) was significantly high in patients with CML compared to the control (*p* < 0.05). The p53 gene serum levels were significantly high in patients with CML (2.135 ± 1.44 ng/mL) compared to the control (0.142 ± 0.11 ng/mL) (*p* < 0.01) (Table 2).

Furthermore, patients with CML treated with either imatinib or nilotinib showed insignificant differences in most of the hematological profile (*p* > 0.05) except in the mean corpuscular hemoglobin, which was high in nilotinib-treated patients compared to imatinib-treated patients (*p* = 0.0002). On the other hand, p53 serum levels were elevated (3.22 ± 1.99 ng/mL) in nilotinib-treated patients and relatively low (1.18 ± 0.19 ng/mL) in imatinib-treated patients (*p* = 0.0001) (Table 3).

## 4. Discussion

In chronic myeloid leukemia, there is an unregulated growth of myeloid and their precursors. CML is a type of myeloproliferative disorder that is associated with a chromosomal abnormality. It accounts for 14% of overall leukemia [13]. Nearly 90% of patients with CML are diagnosed with chronic phase on a routine hematological investigation or may present with abdominal pain due to hepatosplenomegaly, whereas; 10% of patients are diagnosed during the accelerated phase, which is presented as pallor, recurrent infections, and bleeding tendency [14].

In the present study, all patients were in the chronic phase and presented with mild hypochromic microcytic anemia, high white blood cells, low platelet count and platelet mass, compared to healthy subjects. The anemia and low platelet count were due to bone marrow infiltration by clonal myeloid cells and to the peripheral destruction via hepatosplenomegaly. Indeed, 15% of our patients presented with severe hepatosplenomegaly, and this low percentage may explain the mild anemia present in the characteristics of patients [15].

On the other hand, p53 gene serum levels were higher in patients with CML compared to the healthy subjects because, under normal conditions, the p53 gene turnover is preserved at a high level via MDM2 that activates proteosome-mediated p53 gene degradation causing auto-regulatory feedback [16]. As a subsequence to the DNA damage and alteration during the initiation of CML, phosphorylation of MDM2 protein will occur, which stops the inhibitory effect of MDM2 protein on the p53 gene activation. Therefore, the p53 gene will increase but does not enter the cell-cycle untill DNA damage has been restored and thus; high levels of p53 activate apoptosis [17]. This may explain the high level of p53 in patients with CML compared to the control patients. Moreover, the mitochondrial p53 gene is able to activate a pro-apoptotic protein that stimulates the release of cytochrome C causing potential apoptosis [18].

Additionally, the p53 gene performs a depressing effect on the proliferation of myeloid cells via changes in cell kinetics mainly in the chronic phase of CML. In the blast crisis phase, many changes in p53 function will occur due to point mutation, leading to the triggering of a mutant p53 gene that lacks the negative regulatory effect on the myeloid cells [19]. In the present study, all CML patients were in the chronic phase thus, so the p53 gene was non-mutated given the fact that only 20% of CML patients at the blast crisis phase and very little at the chronic phase exhibit mutated p53. Therefore, the stabilization and activation of p53 function via nutlin or MI-219 may augment p53-suppressant effect via inhibition of the interaction between p53 and MDM2 protein during the blast crisis of CML [20].

In the present study, tyrosine kinase inhibitors imatinib and nilotinib increased p53 gene levels in patients with CML compared to healthy subjects which corresponds to Jabbour et al.’s study that illustrated this effective therapy with tyrosine kinase inhibitors for patients with CML [21]. This is because tyrosine kinase inhibitors act through the inhibition of negative regulator proteins on p53 gene functions mainly between p53 and ubiquitin ligase or by inhibiting the binding of ATP to the tyrosine kinase domain of BCR-ABL fusion protein [22].

Imatinib therapy induced p53 gene activation via the inhibition of BCR-ABL and MDM2 translation, so response to the imatinib is correlated with p53 gene levels because imatinib action is p53-dependent [23,24]. This finding corresponds with our results illustrating high p53 levels in imatinib-treated patients compared to the healthy subjects.

Moreover, Henze et al.’s study showed that imatinib modulates p53/MDM2 pathways that improve the apoptotic reaction and response to solid tumors of GIT, so imatinib showed a potent anti-proliferative effect but did not succeed to produce adequate apoptosis resulting in low remission rates [25].

Additionally, Liu et al.’s study illustrated that imatinib might induce the pre-apoptotic pathway independent of p53 activation through activation of specific signal pathways such as mitogen-activated protein kinase p38 (MAPK) and PML-nuclear body. So that p53-independent pathway might be an alternative pathway for the action of imatinib during therapy of patients with CML [26]. This detection may explain the low levels of p53 in imatinib-treated patients compared to nilotinib-treated patients as revealed in our study.

On the other hand, nilotinib which is selective tyrosine kinase inhibitor endorsed for patients with imatinib-resistant CML (since nilotinib therapy produces a lower risk of CML progression) there is a slight possibility of discontinuation and excellent response rate compared to imatinib therapy [27].

Kantarjian et al.’s study revealed that CML patients with imatinib resistance achieved 40% and 28% cytogenic response and molecular response respectively after switching to nilotinib therapy [28], which correspond to our findings since the hematological profile and p53 activation were better in nilotinib-treated patients than imatinib-treated patients.

The mechanisms of resistance to the imatinib therapy may be due to the activation of P-glycoprotein on the malignant cells, augmentation of ABL-BCR expression and ABL-kinase that led to the suboptimal effect and then failure of treatment, but these mechanisms are less evident with nilotinib in view of the fact that it is more potent than imatinib [29]. 

Consequently, 25% of patients with CML developed resistance to imatinib following achievement of complete cytogenic and molecular responses indicating that the response to imatinib may be transient [30].

Finally, tyrosine kinase inhibitors mainly nilotinib produced potential therapeutic effects on the reactivation of the p53 gene in patients with CML as supported by Peterson et al.’s study that illustrated activation of the p53 gene in vitro and in vivo by a specific agent that prevented the binding of the p53 gene with negative regulators regarded as potential pathways for the cure of CML [31].

The current study has limitations. it is a cross-sectional study with a small sample size of no determination of the baseline data on p53 levels or the effect of tyrosine-kinase inhibitors due to the limited amount of related data available.Analysis of gender-related differences and survival rate were not estimated. In spite of these limitations, our study is regarded as a preliminary study for a future prospective study to determine the differential effects of tyrosine-kinase inhibitors on p53 in patients with chronic myeloid leukemia.

## 5. Conclusions 

Nilotinib raises p53 serum levels more effectively than imatinib in in patients with chronic myeloid leukemia. 

## Figures and Tables

**Table 1:** characteristics of the study.

Characteristics	Mean ± SD, N, %
Age (years)	52.67 ± 5.12
Gender (male: female ratio)	33:27:00
Number	88
Patients	60 (68.18)
Control	28 (31.18)
Duration of disease (years)	2.1 ± 1.33
Current chemotherapy	60 (100)
Imatinib	29 (48.33)
Nilotinib	31 (51.67)
Other pharmacotherapy	
Analgesic	43 (71.66)
Antibiotics	51 (85.00)
Antihypertensive drugs	12 (35.00)
Hepatosplenomegaly	
Mild	31 (51.67)
Moderate	22 (36.67)
Huge	9 (15.00)
Smokers	10 (16.66)
Philadelphia chromosome	
Positive	11 (18.33)
Negative	49 (81.67)

Data are expressed as mean ± SD, number and percentage.

**Table 2:** The hematological profile and p53 gene level in patients with CML compared to the healthy subjects.

Variables	Control (*n* = 28)	Patients (*n* = 60)	*p*
Hb (g/L)	14.8 ± 2.63	11.38 ± 3.29	0.21
WBC (×10^9^/L)	6.532 ± 2.4	88.93 ± 18.21	0.000 **
Platelet count (×10^9^/L)	338.68 ± 84.39	101.48 ± 22.57	0.000 **
Plateletcrit (%)	0.24 ± 0.06	0.17 ± 0.04	0.000 **
MPV (fL)	8.4 ± 3.55	14.84 ± 4.94	0.06
RDW (%)	12.72 ± 2.52	11.43 ± 2.99	0.33
RDWCV (%)	15.44 ± 1.78	16.44 ± 2.56	0.04 *
MCH (pg/cell)	29.53 ± 2.64	22.8 ± 3.11	0.35
MCV (fL)	88.31 ± 18.27	86.29 ± 18.39	0.99
MCHC (g/dL)	35.36 ± 1.59	29.86 ± 2.75	0.002 **
P53 (ng/mL)	0.142 ± 0.11	2.135 ± 1.44	0.000 **

Data are expressed as mean± SD; * *p* < 0.05, ** *p* < 0.01; Hb: hemoglobin; WBC: white blood cell; MPV: mean platelet volume; RDW: red blood cell distribution width; RDWCV: red blood cell distribution width coefficient variation; MCH: mean corpuscular hemoglobin; MCHC: mean corpuscular hemoglobin concentration.

**Table 3:** The differential effects of imatinib and nilotinib on the hematological profile and p53 gene level in patients with CML.

Variables	Imatinib (*n* = 29)	Nilotinib (*n* = 31)	t	*p*
Hb (g/L)	11.5 ± 3.69	10.44 ± 3.38	1.15	0.63
WBC (×10 ^9^/L)	87.53 ± 17.4	88.11 ± 18.21	0.126	0.91
Platelet count (×10 ^9^/L)	100.55 ± 22.29	101.99 ± 22.54	−0.24	0.95
Plateletcrit (%)	0.16 ± 0.06	0.17 ± 0.02	−0.85	0.39
MPV (fL)	13.4 ± 3.22	15.52 ± 3.92	−2.29	0.025 *
RDW (%)	11.66 ± 2.92	11.11 ± 2.75	0.75	0.45
RDWCV (%)	15.82 ± 1.74	16.49 ± 2.21	−1.30	0.19
MCH (pg/cell)	19.33 ± 2.74	21.8 ± 2.19	−3.84	0.0002 **
MCV (fL)	88.31 ± 18.13	86.29 ± 16.35	0.45	0.65
MCHC (g/dL)	28.26 ± 1.59	29.11 ± 1.79	−1.94	0.056
P53 (ng/mL)	1.18 ± 0.19	3.22 ± 1.99	−5.58	0.0001 **

Data are expressed as mean ± SD; * *p* < 0.05, ** *p* < 0.01; Hb: hemoglobin; WBC: white blood cell; MPV: mean platelet volume; RDW: red blood cell distribution width; RDWCV: red blood cell distribution width coefficient variation; MCH: mean corpuscular hemoglobin; MCHC: mean corpuscular hemoglobin concentration.
